# Supplementary figures and images for: Passage of human-origin influenza A virus in swine tracheal epithelial cells selects for adaptive mutations in the hemagglutinin gene
Source: PLoS One. 2025 Aug 13;20(8):e0327096. doi: 10.1371/journal.pone.0327096 (PMC12349064; doi:10.1371/journal.pone.0327096)

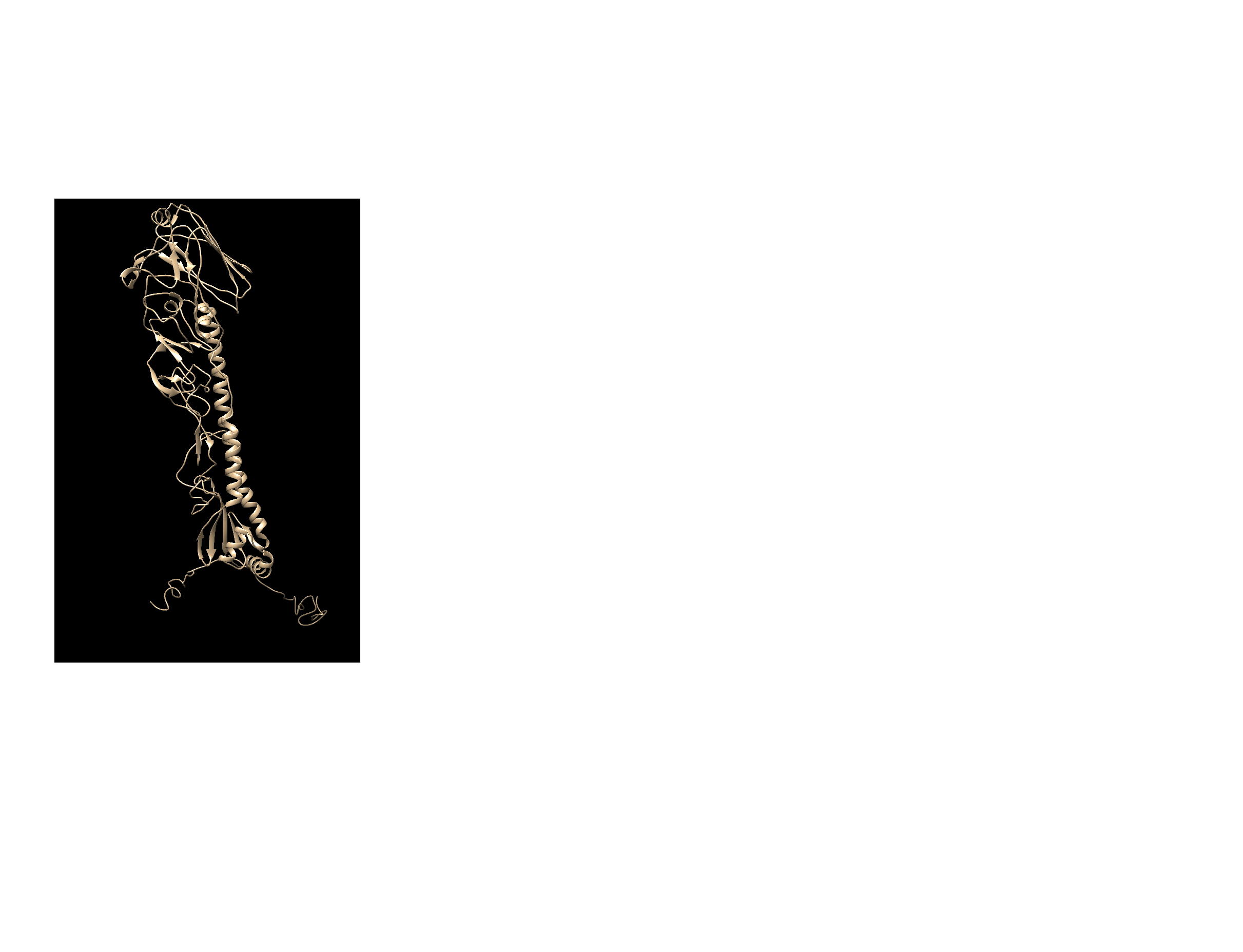

Supplement: S1 File — Protein structure of the H3N2 Hemagglutinin protein. (TIF) [file pone.0327096.s005.tif]
